# Ovarian reserve in women with cystic fibrosis: is this a cause of sub-fertility?

**DOI:** 10.1186/s13048-023-01226-x

**Published:** 2023-07-27

**Authors:** Malena Cohen-Cymberknoh, Keren Marks Garber, Joel Reiter, Michal Shteinberg, Aielet Stolovas, Iyad Barghouti, Chana Adler Lazarovits, Efrat Esh Broder, Anat Hershko Klement

**Affiliations:** 1 Pediatric Pulmonary Unit and Cystic Fibrosis Center, Hadassah Medical Center, Faculty of Medicine, Hebrew University of Jerusalem, Jerusalem, Israel; 2 The IVF Unit, Mount Scopus, Hadassah Medical Center, Faculty of Medicine, Hebrew University of Jerusalem, Jerusalem, Israel; 3 Pulmonology Institute and Cystic Fibrosis Center, Carmel Medical Center and Technion- Israel Institute of Technology and the B. Rappaport Faculty of Medicine, Haifa, Israel; 4 Biochemistry Laboratory, Hadassah Medical Center, Mount Scopus, Jerusalem, Israel; 5 Department of Obstetrics and Gynecology, Faculty of medicine, Hebrew university of Jerusalem, Jerusalem, Israel

**Keywords:** Cystic fibrosis, Anti-mullerian hormone, Subfertility, Infertility, Ovarian reserve, Ovarian follicles

## Abstract

**Background:**

Over the past two decades, increasing number of people with cystic fibrosis (CF) survive into adulthood. Compared to the general population, sub-fertility is an obstacle for many women with CF (wwCF). Decreased ovarian reserve has been proposed as a possible cause, but limited data is available to support this. The aim of this study was to evaluate the ovarian reserve in wwCF and to correlate this with patients’ demographic and clinical data.

**Methods:**

Reproductive-aged wwCF were enrolled during their routine medical appointments. Assessment included Anti-Mullerian hormone (AMH) levels, routine blood tests and antral follicular count (AFC) evaluation. Additionally, demographic, and clinical information were collected.

**Results:**

A total of wenty-three wwCF were enrolled, with ages ranging from 19 to 40 years (median 27 years). Among the fourteen wwCF who were considering pregnancy, five (35.7%) disclosed undergoing an infertility assessment and receiving fertility treatments. All but one patient had an Anti-Mullerian hormone (AMH) level between the 5th and 95th % for age. Measurement of the antral follicular count (AFC) was possible in 12 of the 23 patients and was ranging 8–40 with a median of 17. The proportion of wwCF presenting below median AMH values was not different in sub-fertile as compared to fertile wwCF (P value 0.54). There were no correlations between AMH levels and disease severity parameters. AMH seemed to be relatively higher in wwCF with mild class mutations, but this was not shown to have statistical significance.

**Conclusions:**

Our results, in contrast with the limited available published data, do not support the hypothesis that decreased ovarian reserve plays a major role in infertility in wwCF.

## Background

Cystic fibrosis (CF) is an autosomal recessive disease caused by mutations in the CF trans-membrane conductance regulator (CFTR). It is considered the most common lethal genetic disorder among Caucasians. However, recent advances in understanding CF’s pathophysiology and the development of new treatments, has led to increasing numbers of people with CF who survive into adulthood [1]. The improvement in survival has enhanced quality of life of people with CF, with many forming marital relationships and a desire to have children [2]. Accordingly, there has been a rise in documented pregnancies among women with CF (wwCF) in CF registries [1]. Furthermore, studies have also shown that even wwCF with advanced disease, can carry pregnancies to term, generally resulting in favorable outcomes for both mother and child [3]. Despite these positive trends, a significant proportion of wwCF still face sub-fertility compared with the general population, and the causes of this condition remain diverse and not fully understood [4].

Sub-fertility has been described repeatedly since the 1970s [5], with an array of possible explanations including thick cervical mucus [6, 7], impaired ovulation, abnormal hormonal levels [8], altered uterine bicarbonate secretion [9], and the presence of CFTR in brain areas involved in reproductive functions [10]. While it was initially assumed that mechanical factors, secondary to cervical mucous thickness, were the main cause of wwCF sub-fertility, it is now understood that this is only one factor among many. Decreased ovarian reserve is one such mechanism proposed to explain sub-fertility. Murine studies have shown lower ovarian weights [11] and a single study of young wwCF reported lower anti-Mullerian hormone (AMH) levels, as compared with healthy controls, while mean antral follicular count (AFC) was similar in the two groups [12]. Since only limited data has been published on ovarian reserve, we aimed to evaluate the ovarian reserve in wwCF as measured by anti-Mullerian hormone (AMH) levels and the antral follicular count (AFC) and to study the correlations between ovarian reserve and the patients’ demographic and clinical data.

## Methods

In this cross-sectional study, reproductive age wwCF were recruited during routine follow-up visits at the CF Center in the Hadassah Medical Center, Jerusalem, Israel. All patients included in the study were followed at the same CF Center, ensuring consistency of assessment. The clinic is a tertiary referral center, being addressed by patients from the entire district. Following informed consent, blood was drawn for AMH levels as well as routine CF blood tests. Data on menstrual history, number of pregnancies, births, miscarriages, and a history of subfertility or a need for fertility interventions was collected by a senior gynecologist (AHK). Additionally, a trans-vaginal ultrasound study, to ascertain the AFC in non-virgin patients in early follicular phase. AFC, in a similar manner to AMH, is age dependent, but for all reproductive age sub-groups a value < 5 is considered to represent a diminished ovarian reserve[13, 14]. AMH and AFC were chosen as ovarian reserve markers for the current research due their sensitivity and as complementary tests[13, 15] Demographic and clinical data were collected from the patients’ electronic medical records, including: age, CFTR-mutations (“severe” mutations- 2 mutations from class I-III, or “mild mutations”- at least 1 mutation from class IV or V), body mass index (BMI), pancreatic status [pancreatic insufficiency (PI) or sufficiency (PS)], the presence of CF-related diabetes (CFRD), spirometry values (forced expiratory volume in 1 s (FEV_1_) % predicted, measured according to ATS/ERS guidelines[16]. FEV_1_ results were transformed into Z-scores using the Global Lung Function Initiative calculator (version 3.3.1) and the presence of *Pseudomonas aeruginosa chronic* colonization in sputum (defined by the Leeds criteria, i.e. >50% of the sputum samples over the preceding 12 months) [17]. Laboratory data (complete blood count and C-reactive protein (CRP) were measured, as an indicator of the patients’ inflammatory status. In addition, data regarding medications including novel highly effective modulator therapy (HEMT), and all other routine therapies were recorded.

AMH was measured using the Roche Elecsys® AMH plus assay, on the COBAS 6000 e601 module (Roche Diagnostics), which is an immunoassay for the in-vitro quantitative determination of anti-Mullerian hormone (AMH) in human serum and plasma. Values were referred to the manufacturer reference table, which map out AMH values according to age in a percentile fashion (2.5th, 5th, 50th, and 95th percentile).

All analyses were conducted using SPSS Statistics for Windows, Version 23.0 (IBM Corp., Armonk, NY). All statistical tests were two-tailed and a p-value < 0.05 was considered statistically significant. Quantitative variables were analyzed using the Mann-Whitney test and categorical variables were analyzed using Pearson’s chi-squared test. For statistical comparison purposes the cohort was divided according to CRP values below and above 1 mg/dl, the use of HEMT, FEV_1_ above and below 60% of expected (mild vs. moderate to severe lung disease status [3]), CFRD diagnosis, requirement of vitamin D supplementation, BMI, *Pseudomonas* status, and CFTR-mutation severity class. The study was approved by the local ethics board (HMO-0298-21). Trial registration number NCT05029817 on clinical trials.gov.

## Results

Twenty-three wwCF were enrolled, aged 19–40 years (median of 27 years (Table 1).

**Table 1:** Patients’ demographic characteristics, AMH values and FEV_1_

Serial number	Age (years)	Interest in Childbirth	Age at Menarche	Gravidity	Parity	Living Children	Subfertility*	AMH (ng/ml)	AMH %ile range	BMI	FEV1 (%)
1	20	No	12	0	0	0	N/A	6.2	50–95	24.3	98
2	36	Yes	13	3	1	1	No	2.5	50–95	20.6	60
3	40	No	17	0	0	0	N/A	0.09	5–50	19.9	80
4	19	No	13	0	0	0	N/A	6.47	50–95	20.4	108
5	28	Yes	12	2	2	2	Yes	1.46	5–50	23.7	70
6	27	Yes	13	2	2	2	No	2.61	5–50	21.7	91
7	24	No	15	0	0	0	N/A	1.85	5–50	19.5	82
8	34	Yes	13	3	2	2	Yes	3.19	50–95	21.6	66
9	30	Yes	16	5	4	4	No	0.81	5–50	23.7	79
10	35	No	13	0	0	0	N/A	2.11	50–95	21.6	63
11	27	Yes	13	2	2	3	Yes	1.71	5–50	23.2	75
12	29	Yes	12	2	2	2	No	1.25	5–50	22.4	72
13	24	Yes	13	0	0	0	Yes	0.85	< 5	19.8	59
14	27	No	14	0	0	0	N/A	3.97	50	25.3	107
15	29	Yes	14	3	3	2	No	2.13	5–50	26.1	96
16	29	Yes	13	0	0	0	Yes	3.43	50–95	20	39
17	25	Yes	15	2	2	2	No	3.25	5–50	22.6	109
18	24	Yes	15	0	0	0	No	2.55	5–50	16.9	59
19	22	Yes	12	0	0	0	No	2.55	5–50	23.6	117
20	33	No	13	0	0	0	N/A	1.14	5–50	23.5	71
21	20	No	14	0	0	0	N/A	4.21	50–95	19.5	103
22	22	No	14	0	0	0	N/A	2.33	5–50	24.02	54
23	26	Yes	17	2	2	2	No	4.2	50–95	23.16	112

Abbreviations: AMH (anti-Mullerian hormone); FEV_1_ (forced expiratory volume in 1 s); BMI (body mass index)

Among the fourteen wwCF who were considering pregnancy, five (35.7%) disclosed undergoing an infertility assessment and receiving fertility treatments .Three of these five wwCF had conceived and given birth to one or more children through intra-uterine inseminations. Most of the wwCF (Table 1) had AMH values in the normal range (5-95th percentiles). One woman, aged 24, had an AMH of 0.85, a value below the 2.5th percentile for her age; indeed, she is currently undergoing fertility treatments and has experienced 4 years of infertility since trying to conceive. She has an FEV_1_ of 59% predicted, BMI of 19.76, has CFRD, and had been on HEMT (Elexacaftor/Tezacaftor/Ivacaftor) for three months prior to enrolment and AMH level analysis. Measurement of the AFC was possible in 12 of the 23 wwCF and was ranging 8–40 with a median of 17.

At the time of recruitment, 12/23 participants were on HEMT (three on Tezacaftor/Ivacaftor and nine on Elexacaftor/Tezacaftor/Ivacaftor). We explored correlations between AMH values and various patient characteristics. As shown in Table 2, no statistically significant correlations were found (CRP, HEMT, FEV_1_, diabetic status, vitamine D, BMI, *Pseudomonas aeruginosa* status and mutation severity). Comparing wwCF with mild vs. severe class mutations, AMH seemed to be relatively higher in wwCF with mild class mutations, but this was not shown to have statistical significance.

**Table 2:** AMH values according to CRP categories, novel highly effective CFTR modulators (HEMT) utilization, FEV1 categories, diabetic status, vit D, BMI and genetic traits

Parameter		AMH (ng/ml) Mean ± SD	AMH (ng/ml) median	P value
CRP (mg/dL)	≥ 1	2.3 ± 2.1	1.4	0.177
	< 1	2.8 ± 1.4	2.6	
HEMT	yes	2.5 ± 1.5	2.5	0.695
	no	2.8 ± 1.7	2.3	
FEV_1_	≤ 60%	2.3 ± 0.9	2.5	0.292
	> 60%	2.0 ± 1.5	1.8	
CF related Diabetes	yes	2.4 ± 1.9	1.7	0.378
	no	2.8 ± 1.3	2.5	
Vitamin D supplementation	yes	2.6 ± 1.6	2.5	0.465
	no	3 ± 1.5	3.3	
BMI	< 21	2.7 ± 2	2.5	0.821
	≥ 21	2.6 ± 1.4	2.3	
*Pseudomonas aeruginosa* status	yes	2.2 ± 1.3	2.5	0.406
	no	3.1 ± 1.8	2.3	
Mutation severity class	severe	2.3 ± 1.5	2.4	0.231
	mild	3.2 ± 1.7	3.3	

Abbreviations: AMH (anti-Mullerian hormone); CRP (C-reactive protein); HEMT (highly effective CFTR modulators); FEV_1_ (forced expiratory volume in 1 s); BMI (body mass index)

In addition, we tested whether AMH levels differed by sub-fertility diagnosis: the proportion of wwCF presenting below median AMH values was not different in sub-fertile as compared to fertile patients (P value 0.54).

## Discussion

In our cohort of reproductive age wwCF, all except one woman demonstrated normal AMH levels. The AMH levels range from the 5th to 95th percentile, and 39.1% of wwcF had AMH values at or above the median and preserved AFC, indicating preserved ovarian reserve. We found no correlation between disease severity, serum inflammatory parameters, the use of HEMT, and AMH values. Despite a small number of participants (n = 12) on HEMT, these results are reassuring.

The number of pregnancies in wwCF has increased steadily since the 2000s [1], with a marked increase in the overall pregnancy rate among wwCF aged 14 to 45 years in 2020 compared to 2015, as 619 vs. 235 pregnancies were recorded, respectively. This is especially interesting since the pregnancy rate in the U.S. general population has decreased during this time (according to the CF foundation annual data report of 2020). In our cohort, 10 out of 14 wwCF interested in having offspring, indeed had given birth, with some having been pregnant multiple times. Overall, the patients in our cohort had born twenty-two children, with a range of 1–4 children per woman. It is important to note that four more patients reported additional spontaneous pregnancies, post recruitment and sampling. Data regarding the use of HEMT during pregnancy and lactation is still preliminary, but, as described by Nash et al. [18], it has thus far been reported that these drugs are generally well-tolerated in pregnancy and lactation. Moreover, it has been proposed that because CFTR modulators affect the underlying protein abnormality, which may improve both cervical mucous viscosity and pH, they may therefore improve fertility [19, 20]. Several cases of spontaneous pregnancies in wwCF on HEMT, and a history of infertility have recently been published [21].The MAYFLOWERS study, once complete, will shed light on the effect of CFTR modulators on the pregnancy itself [22].

In the current study, sub-fertility was found in five of fourteen wwCF who attempted pregnancy (35.7%).This finding is consistent with a previous report by Shteinberg et al. [4], which indicated a higher prevalence of sub-fertility in wwCF compared with the general population (35% vs. 5–15% respectively). However, according to our findings, it is unlikely that this sub-fertility is a result of low ovarian reserve. To our knowledge, the assessment of ovarian reserve, specifically measured by AMH and AFC values, has only been previously reported by Schram et al. [12]. In their study, they compared AMH levels between wwCF and health-work controls, and while they found lower AMH levels in the wwCF, both groups had AFC values above 25, which did not correspond with the AMH levels. The authors concluded that the lower AMH levels suggest a diminished ovarian reserve and recommended considering assisted reproductive techniques for infertile wwCF. However, the AMH values in the control group were higher than average suggesting that the control group may have failed to represent the general population accurately; furthermore, the AMH levels of the wwCF were mostly in the normal range for age, as were the high AFC levels, supporting preserved ovarian reserve in these patients. In the current study, we compared the results of our cohort to established reference values and found that the majority of our patients fell within the normal range.

These findings provide important and supportive information regarding ovarian reserve in wwCF, aligning with the reproductive capabilities demonstrated by our patients and the recorded measures of AFC. This understanding of reproductive performance in wwCF can have positive psychosocial effects, influencing how CF patients perceive their overall health and potentially reducing the stigma associated with family planning among wwCF.

Our study has several limitations that should be acknowledged. Firstly, although this study represents the largest group published thus far regarding AMH levels in wwCF, the cohort size remains modest, consisting of 23 wwCF. Furthermore, it is important to note that our cohort primarily comprises young women who are often religious, with a significant proportion being ultraorthodox. This demographic factor may influence their reproductive outcomes, as they tend to marry and conceive at a relatively young age, possibly contributing to the positive reproductive results, as observed in the present study. Longer follow-up of these findings would be beneficial in providing more comprehensive insights into fertility in wwCF. Finally, although the results are reassuring regarding the safety of HEMT, it is important to highlight that this study was not specifically designed as a safety study. Therefore, caution should be taken when interpreting these results, and further dedicated research is necessary to fully evaluate the safety aspects of HEMT in wwCF.

## Conclusions

To conclude, according to our study, and contrary to the limited data currently available in the literature, the majority of wwCF do not experience diminished ovarian reserve. This novel insight has implications for the approach to fertility in wwCF. However, further research is required to comprehensively understand the underlying causes of impaired fertility in wwCF and to explore the potential influence of HEMT on fertility outcomes. Continued investigation in these areas will contribute to a more comprehensive understanding of reproductive health in wwCF.

## Data Availability

The datasets used and/or analyzed during the current study are available from the corresponding author on reasonable request.

## Abbreviations

CF: Cystic Fibrosis
wwCF: women with CF
AMH: anti-Mullerian hormone
AFC: antral follicular count
CFTR: Cystic fibrosis transmembrane conductance regulator
BMI: body mass index
PI: pancreatic insufficiency
CFRD: CF-related diabetes
FEV_1_: forced expiratory volume in 1 s
CRP: C-reactive protein
HEMT: novel highly effective CFTR modulators
